# Government Hospitals for the Consumptive Poor

**Published:** 1900-07

**Authors:** F. Paschal

**Affiliations:** San Antonio, Texas


### Government Hospitals for the Consumptive Poor.

Letter from Dr. Paschal, Health Officer of San Antonio.

SAN ANTONIO, TEXAS, June 14, 1900.

*Editor Texas Medical Journal:*

I am engaged in trying to excite interest in having the U. S. government build institutions to care for those poor helpless tubercular people who start on pilgrimages seeking health, without means and without friends, being furnished with a pass to the next nearest town, a bucket of food and enough creosote to stink the car out and told to "hunt climate." You know the rest; they land in some hospital, poor house, or perhaps jail, and there stay until their wasted forms perish and their spirit takes its flight to that abode, whence pilgrims never return. This turning loose on communities of helpless invalids is to me a sad reflection upon advanced civilization, and I think it is time for this great humane government to open its eyes to the importance of taking charge of these poor people, not alone for charity sake, but on account of isolating them and preventing the spread, so far as possible, of the "white plague." Until such measures are adopted we will continue to have one-sixth of our deaths due to tuberculosis. Desperate measures are used to prevent the bubonic plague from getting to our shore. But with regard to tuberculosis, which is *endemic* and which carries off hundreds of thousands of victims, the greatest placidity is shown by our government. I hope you will take this matter up and send your lance deep into the subject and let us see if it is not possible that early years of the twentieth century may witness the creation of magnificent institutions in salubrious parts of this country for the care of helpless, poor consumptives.

I hope to live to see the U. S. government assume charge of them.

F. PASCHAL, M. D.

